# Bioenergetic Analysis of Ovarian Cancer Cell Lines: Profiling of Histological Subtypes and Identification of a Mitochondria-Defective Cell Line

**DOI:** 10.1371/journal.pone.0098479

**Published:** 2014-05-23

**Authors:** Usawadee Dier, Dong-Hui Shin, L. P. Madhubhani P. Hemachandra, Larissa M. Uusitalo, Nadine Hempel

**Affiliations:** Nanobioscience Constellation, College of Nanoscale Science and Engineering, State University of New York, Albany, New York, United States of America; University of Alabama at Birmingham, United States of America

## Abstract

Epithelial ovarian cancer (EOC) is the most lethal of all gynecological cancers, and encompasses distinct histological subtypes that have specific genetic and tissues-of-origin differences. Ovarian clear cell carcinoma (OCCC) represents approximately 10% of cases and has been termed a stress responsive cancer. OCCC is characterized by increased expression of oxidative stress and glycolysis-related genes. In the present study, we hypothesized that bioenergetic profiling might uniquely distinguish OCCC from other EOC histological subtypes. Using an extracellular flux analyzer, OCCC lines (ES-2, TOV-21-G) were shown to be highly metabolically active, with high oxygen consumption rate (OCR) and high extracellular acidification rate (ECAR), indicative of enhanced mitochondrial oxidative phosphorylation and glycolytic rate, respectively. A high bioenergetics profile was associated with the cell lines' ability to form anchorage independent spheroids. Given their high glycolytic and mitochondrial activity, OCCC cells displayed strong sensitivity to 2-deoxy-D-glucose and Rotenone growth inhibition, although this chemosensitivity profile was not specific to only OCCC cells. Bioenergetic profiling also identified a non-OCCC cell line, OVCA420, to have severely compromised mitochondrial function, based on low OCR and a lack of stimulation of maximal respiration following application of the uncoupler FCCP. This was accompanied by mitochondrial morphology changes indicative of enhanced fission, increased expression of the mitochondrial fission protein Drp1, a loss of mitochondrial membrane potential and dependence on glycolysis. Importantly, this loss of mitochondrial function was accompanied by the inability of OVCA420 cells to cope with hypoxic stress, and a compromised ability to stabilize HIF-1α in response to 1% O_2_ hypoxia. This knowledge may be imperative for researchers planning to utilize this cell line for further studies of metabolism and hypoxia, and suggests that altered mitochondrial fission dynamics represents a phenotype of a subpopulation of EOCs.

## Introduction

Ovarian cancer remains one of the deadliest cancers in women, with little improvement in overall survival reported over the last three decades. It has become apparent that ovarian cancer is a broad term used for a number of distinct diseases, sharing the same anatomical location within the intraperitoneal (IP) cavity. The five subtypes of epithelial ovarian cancer (EOC) differ significantly in their tissue of origin, genomic markers and reliance on different pro-tumorigenic cell signaling pathways [1]–[3]. High-grade serous ovarian cancer (SOC) is the most common histological subtype and characterized by high frequency in TP53 mutations, genomic instability and as being of fallopian tube origin [3], [4]. Ovarian clear cell carcinomas (OCCC) represent approximately 10% of EOC cases in western populations (up to 25% in Asian populations) [5]. OCCCs appear to consist of heterogeneous subpopulations displaying various degrees of genomic aberrations [6]. The most common are associated with the AT-rich interacting domain containing protein 1A (ARID1A mutation ∼50%) [7], [8] and the PI3K pathway (PTEN loss ∼40% [9], PIK3CA mutation [10]; AKT2 amplification [5]). ARID1A mutations have allowed researchers to associate early OCCC lesions with endometrioid tissues and endometriosis cysts [8], [11].

While there are significant differences in genomic aberrations between individual OCCC specimen, Yamaguchi and colleagues have recently reported a gene expression signature that is uniquely associated with OCCC [12]. In particular, this study reconfirmed other reports that OCCC is characterized as a stress responsive cancer [12]–[14]. High expression of antioxidant enzymes and genes associated with glucose metabolism are also prevalent [12], [15]. This expression profile is thought to represent adaptations of OCCC against stressors of the tumor microenvironment, including free-iron induced redox stress and inflammation [16]. Some of these expression changes are similarly observed in endometrial cysts, further suggesting that this represents the precursor tissue of OCCC [12].

While early stage OCCC patients generally have a better survival rate than early stage SOC patients, stage III and IV OCCC is associated with poor survival. In addition, less than 10% of recurrent OCCC respond to therapy and this histological subtype has been associated with high cisplatin-resistance [5]. Given that there are significant differences in the OCCC genome and expression profile compared to SOC, there is a need to further understand the molecular mechanisms that drive OCCC tumorigenesis and progression to tailor therapeutics for this particular histological subtype. Given that OCCCs are characterized by high expression of mediators of the glycolytic pathway, the aim of the present study was to investigate if OCCC cell lines also significantly differ in their bioenergetics profile compared to other EOC cells in culture. Using live cell measurements of oxygen consumption and extracellular acidification, we were able to establish that OCCC cell lines are highly metabolic. In addition, this analysis revealed a SOC cell line with defective mitochondrial function, which manifested in a loss in hypoxic response of these cells.

## Materials and Methods

### Cell Lines

OVCA420, OVCA429, OVCA433, DOV-13 and NOSE007 cells were generously provided by Dr. Susan K. Murphy (Duke University) and cultured in RPMI1640 containing 10% fetal bovine serum (FBS). OVCA420, OVCA429, OVCA433, DOV-13 cells were originally isolated from ovarian cancer patient ascites [17], [18]. NIHOVCAR3 cells (referred here as OVCAR3) and ovarian clear cell carcinoma cell lines ES-2 and TOV-21-G were purchased from ATCC. ES-2 cells were cultured in Modified McCoy's 5a Medium with 10% FBS. TOV-21-G cells were cultured in a 1∶1 mix of MCDB 105 containing 1.5 g/L sodium bicarbonate and Medium 199 containing 2.2 g/L sodium bicarbonate, with 15% fetal bovine serum. OVCAR3 cells were maintained in RPMI1640, containing 0.01 mg/ml bovine insulin and 20% FBS. Glutamine and Glucose deprivation was carried out using MP Biomedicals RPMI 1640 Medium, 1X Liq., w/o L-Glutamine and Glucose.

### Bioenergetic Analysis of Oxygen Consumption Rate (OCR) and Extracellular Acidification Rate (ECAR)

The Seahorse XF24_3_ Extracellular Flux Analyzer (Seahorse Bioscience, North Billerica, MA) was used to assess bioenergetic phenotypes of ovarian cancer cell lines. The Extracellular Flux Analyzer enables simultaneous live cell measurement of oxygen consumption rate (OCR), an indicator of mitochondrial respiration, and extracellular acidification rate (ECAR), an indicator of net proton loss during glycolysis. Prior to the start of the experiment, cells were evenly seeded (40,000 cells/well; following optimization of cell seeding number) into the XF24 cell culture plate and allowed to attach for 24 hours. Cell culture media was replaced with XF media (Seahorse Bioscience), lacking sodium bicarbonate and FBS, after prior washing with XF media. Cells were placed in a non-CO_2_ 37°C incubator for 1 hour, prior to start of the experiment. OCR and ECAR was measured over a 3 minute period, followed by 3 min mixing and re-oxygenation of the media. Three basal rate measurements were taken prior to injection of pharmacological manipulators of mitochondrial respiratory chain proteins, otherwise known as the Mitochondrial stress test. Several bioenergetics parameters can be deduced by monitoring OCR in response to these compounds [19]. Following the establishment of a basal OCR reading, Oligomycin A is applied to inhibit proton (H+) flow through ATP synthase, essentially blocking all ATP-linked oxygen consumption. Maximal respiration can be initiated by exposing cells to Carbonyl cyanide-ptrifluoromethoxyphenyl hydrazone (FCCP), which is an ionophore that transports H+ across the mitochondrial membrane leading to collapse of membrane potential and rapid consumption of O_2_. Antimycin A prevents mitochondrial respiration by blocking complex III (Ubiquinone:Cytochrome b-c complex). ATP synthase inhibition was achieved by injecting 1 µM Oligomycin A (Sigma), which was followed by injection of 750 nM FCCP (Sigma) for measurement of maximal OCR. This was followed by addition of 1 µM Antimycin A (Sigma) to shut down all mitochondrial respiration. Three measurements of OCR/ECAR were obtained following injection of each drug and drug concentrations optimized on cell lines prior to experiments. OCR and ECAR readings were normalized to total protein levels (BCA protein assay, Pierce) in each well. Each cell line was represented in 5 wells per experiment and replicate experiments carried out at least three times. Basal respiration was derived by subtracting the third OCR reading, following Antimycin A addition, from the third basal OCR reading. Respiratory State apparent, an indication of the mitochondrial respiratory state, was calculated using: 4−(Basal OCR−Oligo OCR)/(FCCP OCR−Oligo OCR) [20]. Prior to commencement of these experiments dose response curves (100 nM–3 µM) were carried out to establish the concentration of FCCP needed to achieve maximal OCR. All cell lines responded by achieving a plateau of maximal OCR at 750 nM FCCP, with inhibition of OCR observed at 3 µM in some cell lines (OVCA429, Ovca433, Ovcar3, ES-2). OVCA420 cells did not respond to FCCP at any of the concentrations tested.

### Real time semi quantitative RT-PCR

Following RNA isolation (RNeasy Mini Kit; Qiagen) and reverse transcription using iScript cDNA Synthesis (BioRad), real time semi-quantitative RT-PCR was carried out on an Applied Biosystems 7500 Real Time PCR cycler, using iTaq Universal SYBR Green Supermix (Bio-Rad). The following primer pairs were used for semi-quantitative real time RT-PCR analysis: HNF-1β sense: 5′-GCCCACACACCACTTACTTCG-3′; HNF-1β antisense, 5′-GTCCGTCAGGTAAGCAGGGAC-3′ [21]. GLUT-1 sense 5′-CATCCTTATTGCCCAGGTGTTT-3′; GLUT-1 antisense 5′-GAAGACGACACTGAGCAGCAGA-3′ [22]. Data were analyzed using the comparative CT method with values normalized to β-Actin levels and expressed relative to levels in NOSE007 cells.

### Spheroid Formation Assay

Ovarian cancer cell lines were seeded (1000 cells/well) into 96 well round bottom ultra-low attachment (ULA) plates (Corning) and monitored for spheroid aggregate formation up to Day 9 under normal culture conditions (fully supplemented media, 37°C, 21% O_2_, 5% CO_2_).

### Cell viability assays

Equal numbers of cells were seeded into 96 well plates and cell viability in response to chemotherapeutic agents measured following 72 hours of drug treatment with indicated doses. Cell viability was assessed by crystal violet uptake. Briefly, cells were stained with crystal violet for 10 minutes, followed by washing in PBS and H_2_O. Crystal violet dye was released from cells using 30% acetic acid and absorbance measured at 590 nm, using a SpectraMax Paradigm Molecular Devices microplate reader. Cell viability was expressed as percentage viability relative to non-treated cells. Cisplatin (cis-Diamineplatinum(II) dichloride), Paclitaxel and 2-deoxy-D-glucose (2-DG) were purchased from Sigma. Rotenone was obtained from Seahorse Biosciences.

### Mitotracker staining

Cells cultured on coverslips were incubated with 250 nM Mitotracker Red CMX ROS (Life Technologies) for 15 minutes, followed by washing in PBS and fixation with 4% paraformaldehyde. Samples were mounted onto slides using Prolong Gold/DAPI (Life Technologies) and imaged using an eVOSfl AMG LED-based fluorescent microscope (100x oil immersion objective).

### mtDNA/nDNA ratio real time RT-PCR

For quantifying mitochondria DNA (mtDNA) and nuclear DNA (nDNA) total DNA from ovarian cancer cells was extracted using AllPrep DNA/RNA/Protein Mini Kit (Qiagen), which was diluted to a final concentration of 10 ng/µl. To quantify the relative mtDNA:nDNA ratio the following primers were used; 16S rRNA gene of mitochondrial (mt)DNA (sense: 5′-CCAAACCCACTCCACCTTAC-3′; antisense: 5′-TCATCTTTCCCTTGCGGTA-3′); 18S rRNA of nuclear (n)DNA (sense: 5′-AGAAACGGCTACCACATCCA-3′, antisense: 5′-CACCAGACTTGCCCTCCA-3′) [23], [24]. CT values for mt16S and n18S were obtained following Real time PCR of 50 ng total DNA using iTaq Universal SYBR Green Supermix (Bio-Rad), at an annealing temperature of 59°C.

### Mitochondrial Membrane potential

Mitochondrial membrane potential was measured using JC-1 dye (5′,6,6′-tetrachloro-1,1′,3,3′-tetraethylbenzimidazolylcarbocyanine iodide; Life Technologies), according to manufacturer's instructions. Briefly, cells were incubated with 10 µg/ml JC-1 dye for 15 min and fluorescence images taken using a 20x objective. The ratio of red fluorescence JC-1 aggregates and green JC-1 monomers was measured using image J, following image background correction.

### Clonogenicity

Cells were seeded (100 cells/well) into a 6 well plate and cultured for 10 days under normal culture conditions (21% O_2_) or hypoxia (1% O_2_, Biospherix hypoxic chamber). Clonogenicity was assessed by staining colonies with crystal violet and number of colonies counted using Image J and data expressed as cellular survival fraction.

### Immunoblotting

Cell lines were cultured in 10 cm dishes to sub-confluency and lyzed in RIPA buffer (+ protease inhibitors), followed by electrophoresis on 4-12% SDS-PAGE gels (Bio-Rad). Following western transfer, membranes were probed with primary Anti-Drp1 antibody (Millipore) or GAPDH (Applied Biosystems-Life Technologies) overnight in 5% non-fat milk/TBS, 0.1% Tween20. Following secondary antibody incubation blots were visualized using Femto or Pico chemiluminescence substrate (Thermo Scientific). For HIF-1α analysis, cells were cultured under hypoxic conditions (1% O_2_) for two hours. Cells were immediately lysed in 2x SDS sample buffer and equal amounts loaded on SDS-PAGE (4–12%, Bio-Rad), followed by western transfer and immunoblotting for HIF-1α and β-actin loading control. Anti-HIF-1α antibody was purchased from BD Transduction Laboratories and β-actin antibody from Applied Biosystems-Life Technologies.

### Data and Statistical Analysis

All data presented are representative of at least three replicate experiments. Image J was used to quantify relative fluorescence and measure spheroid area size. All data are represented as mean ± standard error of the mean. Statistical data analysis (ANOVA with Tukey's post test, or T-test) was carried out using GraphPad Prism Software (v6), and data considered significant at p>0.05.

## Results

### Respiratory Profile of Ovarian Cancer Cell Lines

To characterize the bioenergetics profile of ovarian cancer cell lines we utilized an extracellular flux analyzer to determine live cell oxygen consumption rate (OCR) and extracellular acidification rate (ECAR; Seahorse Bioscience). Two OCCC cell lines, ES-2 and TOV-21-G were compared to non-OCCC EOC cell lines (OVCA420, OVCA429, OVCA433, DOV-13, OVCAR3) and a normal ovarian surface epithelial cell line (NOSE007).

**Figure 1:**
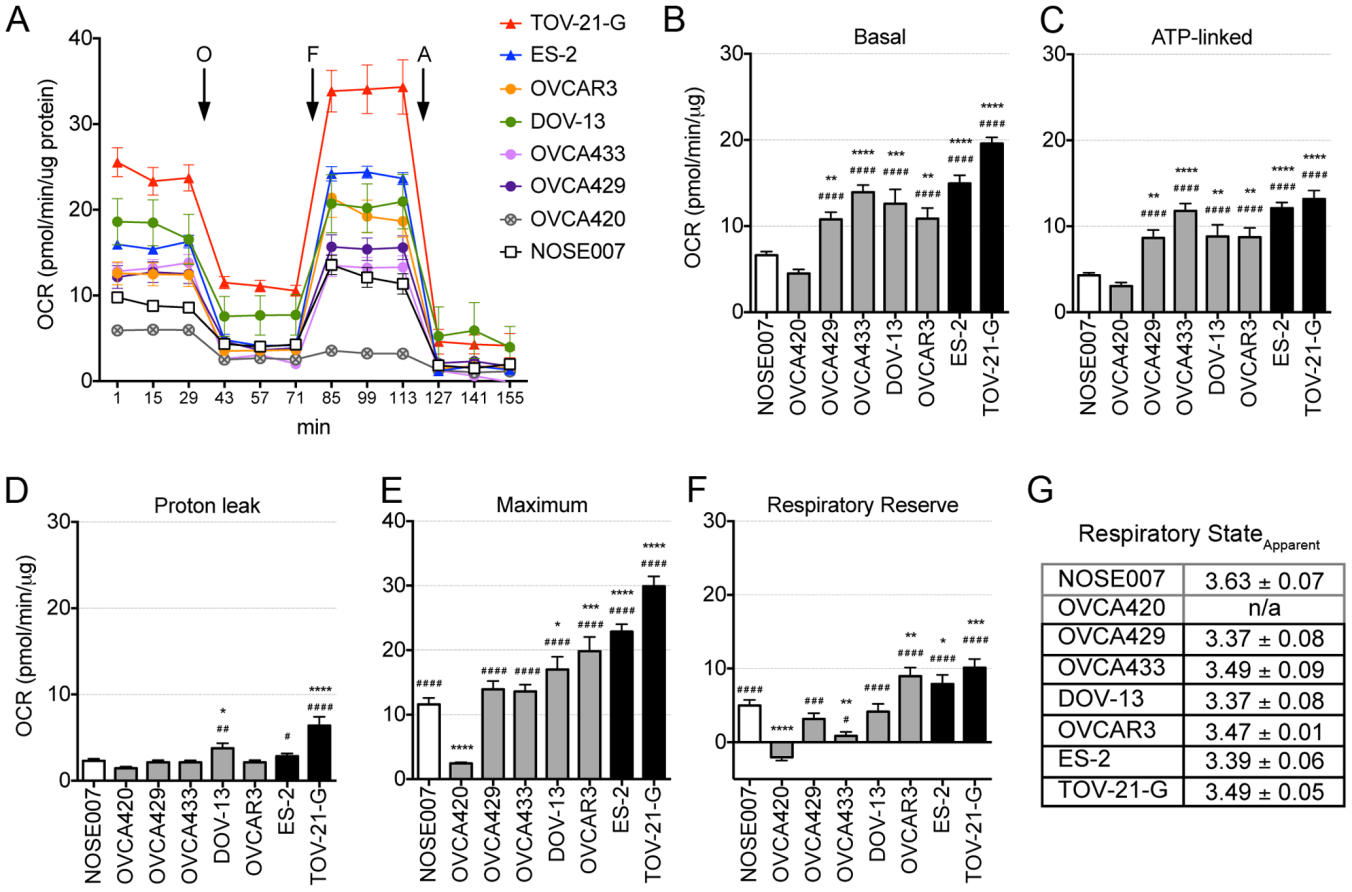
Mitochondrial Respiratory Profile of ovarian cancer cell lines. **A**. Oxygen Consumption Rate (OCR) measurements were obtained over time (min) using an extracellular flux analyzer (Seahorse Bioscience). The mitochondrial stress test was used to obtain bioenergetics parameters, by adding the ATP synthase inhibitor Oligomycin A (O, 1 µM), to derive ATP-linked OCR, FCCP (F, 750 nM) to uncouple the mitochondria for maximal OCR, and Antimycin A (A, 1 µM). **B**. Basal mitochondrial OCR of ovarian cancer cell lines was derived by subtracting non-mitochondrial OCR (remaining OCR after Antimycin A addition). **C**. ATP-linked OCR was derived as the difference between basal and Antimycin A inhibited OCR. **D**. OCR attributed to proton leak was calculated as the difference between OCR following Oligomycin A inhibition and OCR following Antimycin A inhibition. **E**. Maximum OCR was stimulated by FCCP addition. **F**. The respiratory reserve capacity was calculated as the difference between maximal and basal OCR. Graphs B-F represent data from one replicate experiment (n = 5, ANOVA, Tukey's post test *p<0.05, **p<0.01, ***p<0.001, ****p<0.0001 statistically significant compared to NOSE007; #p<0.05, ##p<0.01, ###p<0.001, ####p<0.0001 statistically significant compared to OVCA420). OCCC cell lines are highlighted in black and a normal epithelial cell line in white. **G**. Average Respiratory State_Apparent_ was calculated from 3–4 replicative experiments using: 4−(Basal OCR−Oligo OCR)/(FCCP OCR−Oligo OCR).

**Figure 1A** shows the OCR of cell lines in response to compounds used in the mitochondrial stress test over time. Basal and ATP-dependent OCR readings indicated that most of the cancer cell lines tested, with the exception of OVCA420 cells, had significantly higher OCR compared to a normal ovarian surface epithelial cell line (NOSE007, **Figures 1B&C**). The NOSE007 cell line was not chosen as a representative for the tissue of ovarian cancer origin, as these greatly vary between histological subtypes, but rather as a representative of a normal epithelial cell line. Basal mitochondrial OCR readings (**Figure 1B**) were normalized by subtracting non-mitochondrial OCR, which was low and not significantly different among the cell lines tested (not shown). Most of the Basal mitochondrial OCR was dedicated to ATP-production, as indicated by Oligomycin A inhibition (**Figure 1C**). Any remaining OCR can be somewhat attributed to proton leakage across the membrane [19], which was similar in all cell lines tested, except TOV-21-G and DOV-13 cells, which displayed a slight but significant increase compared to NOSE007 and OVCA420 cells (**Figure 1D**). All cell lines, except OVCA420 cells responded to FCCP by increasing their OCR to maximal rates (**Figure 1E**). Hence, OVCA420 cells did not have an appreciable respiratory reserve capacity, the difference between maximal and basal respiration (**Figure 1F**). High respiratory reserve capacity is also linked to high mitochondrial fidelity. Calculations of the Respiratory State_Apparent_ showed that normal ovarian epithelial cells NOSE007 are operating at submaximal respiratory capacity compared to the ovarian cancer cell lines (**Figure 1G**). True state 4 respiration is characterized by a lack of mitochondrial ATP generation, as in the case of ADP depletion, and mimicked in our assay by Oligomycin A addition. State 3 isolated mitochondria are characterized by maximal activity in the presence of unlimited substrate and ADP [20]. While these parameters are never fully achieved in the context of a cell (cellular State_Apparent_ is therefore considered to be 3.5), we observed that cancer cells, except OVCA420s, displayed a state apparent below 3.5, whereas the State_Apparent_ values for NOSE007 cells approached state 4 (**Figure 1G**). No State_Apparent_ could be obtained for OVCA420 cells due to a lack in response to FCCP.

While all cancer cells clearly had high mitochondrial respiration compared to NOSE007 cells we did not observe a significant difference in respiratory parameters between the OCCC cells and other ovarian cancer cell lines, suggesting that mitochondrial oxygen consumption profile does not differentiate ovarian cancer histological subtypes. However, using this approach we were able to identify OVCA420 cells as having defective mitochondrial function, indicated by low basal OCR and a lack of response to FCCP.

### OCCC cells have high extracellular acidification rate

**Figure 2:**
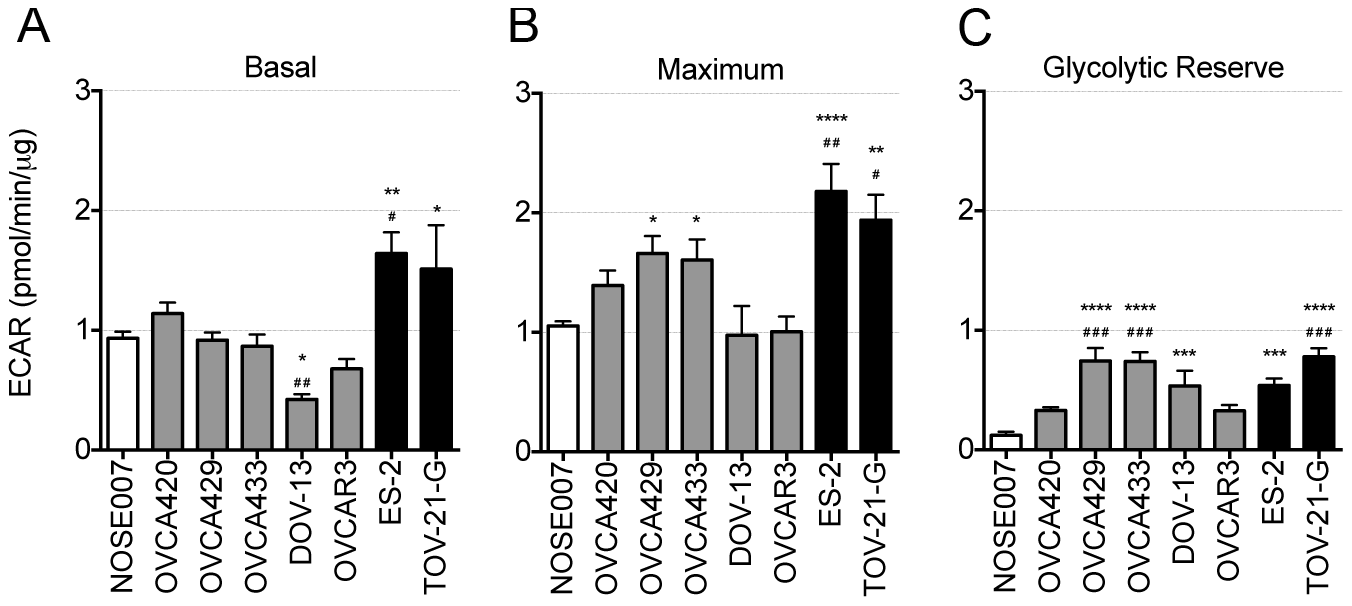
Extracellular Acidification Rate (ECAR) profile of Ovarian Cancer Cell lines. **A**. Basal ECAR levels were measured using an extracellular flux analyzer (Seahorse Bioscience). **B**. Maximal ECAR was stimulated by addition of 1 µM Oligomycin A. **C**. Glycolytic Reserve Capacity was calculated as the difference between maximal and basal OCR. Data from one replicative experiment is shown (n = 5, ANOVA, Tukey's post test *p<0.05, **p<0.01, ***p<0.001, ****p<0.0001 statistically significant compared to NOSE007; #p<0.05, ##p<0.01, ###p<0.001 statistically significant compared to OVCA420).

Simultaneous to measuring OCR, extracellular flux analysis allows for assessment of extracellular acidification rate (ECAR) of culture media. This is considered an indirect analysis of the glycolytic rate of cells [19]. OCCC cell lines ES-2 and TOV-21-G displayed high basal ECAR, whereas other cell line had lower ECAR, similar to NOSE007 (**Figure 2A**). Oligomycin A was used to stimulate maximal ECAR, which shuts down ATP-dependent OCR, effectively shifting metabolism from oxidative phosphorylation to glycolysis (**Figure 2B**). The difference between maximal and basal ECAR is considered the glycolytic reserve capacity of cells (**Figure 2C**). OVCA420 cells, which displayed defective mitochondrial respiration (**Figure 1**), also had low glycolytic reserve capacity, indicating these cells are operating close to their maximal glycolytic rate as a compensation for loss of OCR. This suggests a reliance on glycolysis by OVCA420 cells. The normal epithelial cell line NOSE007 displayed the lowest glycolytic reserve, suggesting that their capacity for glycolysis is lower than that of the other cancer cell lines.

### Bioenergetics Profile

**Figure 3:**
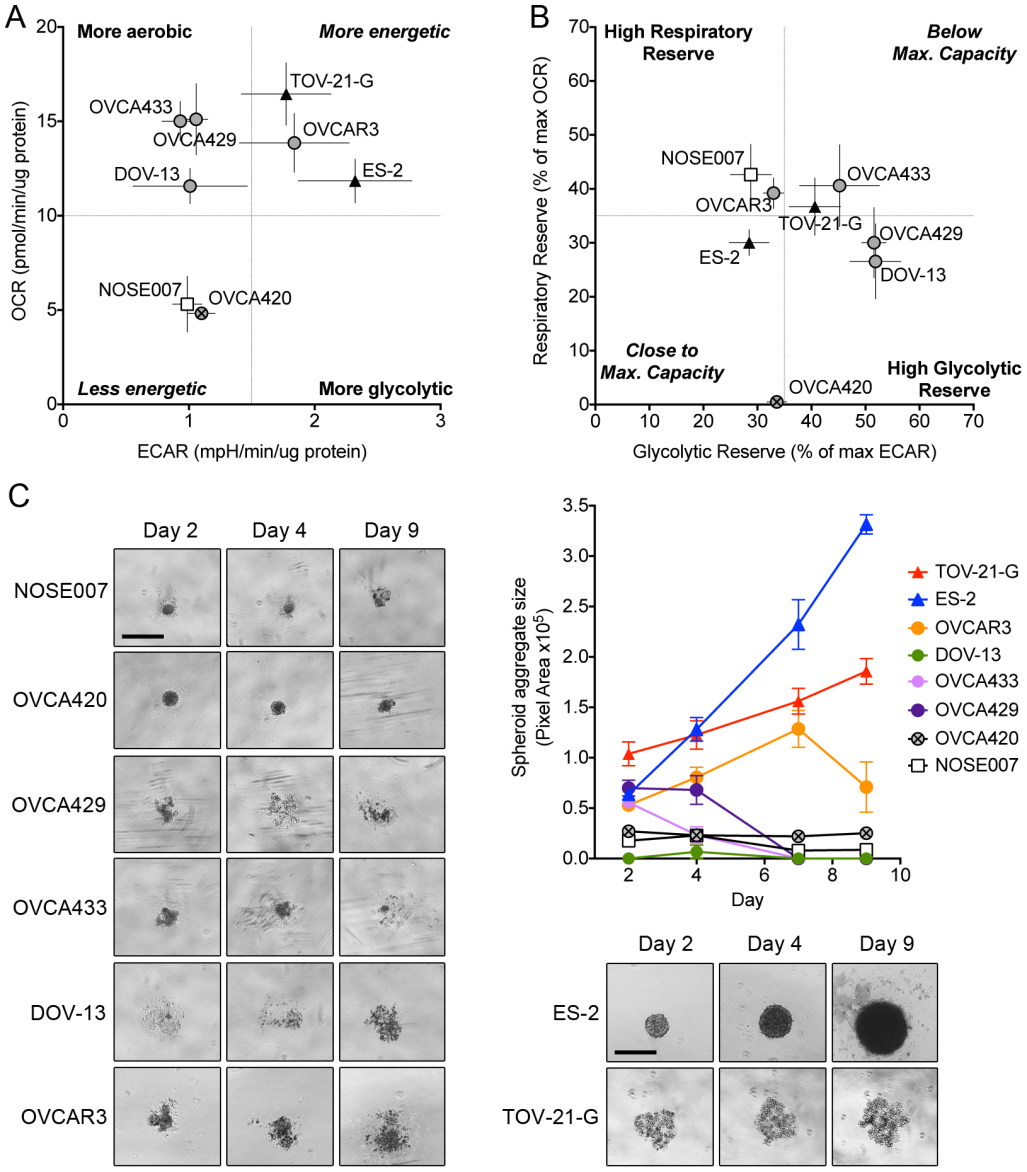
Bioenergetics Profile of Ovarian Cancer cell lines. **A**. Plotting Basal ECAR and OCR levels provides a snap-shot of the bioenergetics profiles of the ovarian cancer cell lines studied. OCCC cell lines ES-2 and TOV-21-G as well as OVCAR3 cells display high glycolysis and Oxidative phosphorylation and are therefore classified as highly energetic cells. **B**. Plotting Glycolytic Reserve against Respiratory reserve indicates that some cells appear to be operating close to their maximal rate, such as ES-2 cells, whereas others have high respiratory reserve (NOSE007) or high glycolytic Reserve (DOV-13, OVCA429). OCCC cell lines are labeled as black triangles, other EOC cell lines as grey circles (OVCA420 as circle with X), and the normal epithelial cell lines NOSE007 as a white square. Data in A and C represents the average of mean OCR and ECAR readings from 3–4 experimental replicates **C**. Spheroid formation correlates with bioenergetic signature of ovarian cancer cells. Cells were seeded into ULA plates and size (area of spheroid aggregate in pixels) plotted (n = 6).

To obtain a better overall picture of the bioenergetics profile of the ovarian cancer cell lines studied we plotted basal ECAR against mitochondrial OCR (**Figure 3A**). This clearly demonstrated how the different cell lines fall into several bioenergetics categories. We found 3 distinct groups of cellular bioenergetics profiles. OCCC cells TOV-21-G and ES-2 clearly represent highly energetic cells with high respiration and glycolysis. OVCAR3 cells similarly displayed this pattern. OVCA433, OVCA429 and DOV-13 had a more aerobic phenotype, while NOSE007 and OVCA420 cells were the least energetic cells, with both low OCR and ECAR. It should be noted that unlike OCR, the ECAR measurements were more variable between experimental days, which was particularly evident for OVCAR3 and DOV-13 cell lines (**Figure 3A**). Interestingly, the more aerobic cells OVCA433, OVCA429 and DOV-13 had a relatively high glycolytic reserve, suggesting that these cells are able to ramp up their glycolytic activity when needed, as may be the case under hypoxic conditions (**Figure 3B**). ES-2 cells displayed the lowest reserve capacities, indicating that this cell line may be operating close to its maximal respiratory and glycolytic capacity under normal culture conditions (**Figure 3B**). When considering both OCR and ECAR parameters, our data suggest that OCCC cells are highly energetic, relying on both oxidative phosphorylation and glycolysis to meet their energy needs. However, respiratory and glycolytic profiles may not be sufficient to differentiate OCCC cells from other histological subtypes, as OVCAR3 cells share similar bioenergetics characteristics.

The ability to survive as anchorage-independent spheroid aggregate plays a large role in the transcoelomic route of ovarian cancer metastasis through the IP cavity. We therefore assessed whether a high bioenergetics profile provides an advantage in spheroid formation and survival. Interestingly, highly energetic cells, ES-2, TOV-21-G and OVCAR3 were able to form and maintain large spheroid aggregates when seeded onto ultra-low attachment surfaces (**Figure 3C**). While OCCC spheroids continued to increase in size, OVCAR3 aggregates started to disaggregate at Day 9. Cells with a more aerobic phenotype were either unable to form spheroids (DOV-13) or to maintain aggregates under anchorage-independent growth conditions beyond day 4 (OVCA429 & OVCA433). NOSE007 and OVCA420 were able to form very small spheroid aggregates, which did not increase in size and started to disintegrate after day 4 and day 9, respectively. These data indicate that a high bioenergetics signature (high OCR and ECAR) may be associated with the ability to form spheroids, potentially aiding anoikis-resistance and anchorage-independent cell proliferation.

### Relationship of Bioenergetics to Chemosensitivity profiles

**Figure 4:**
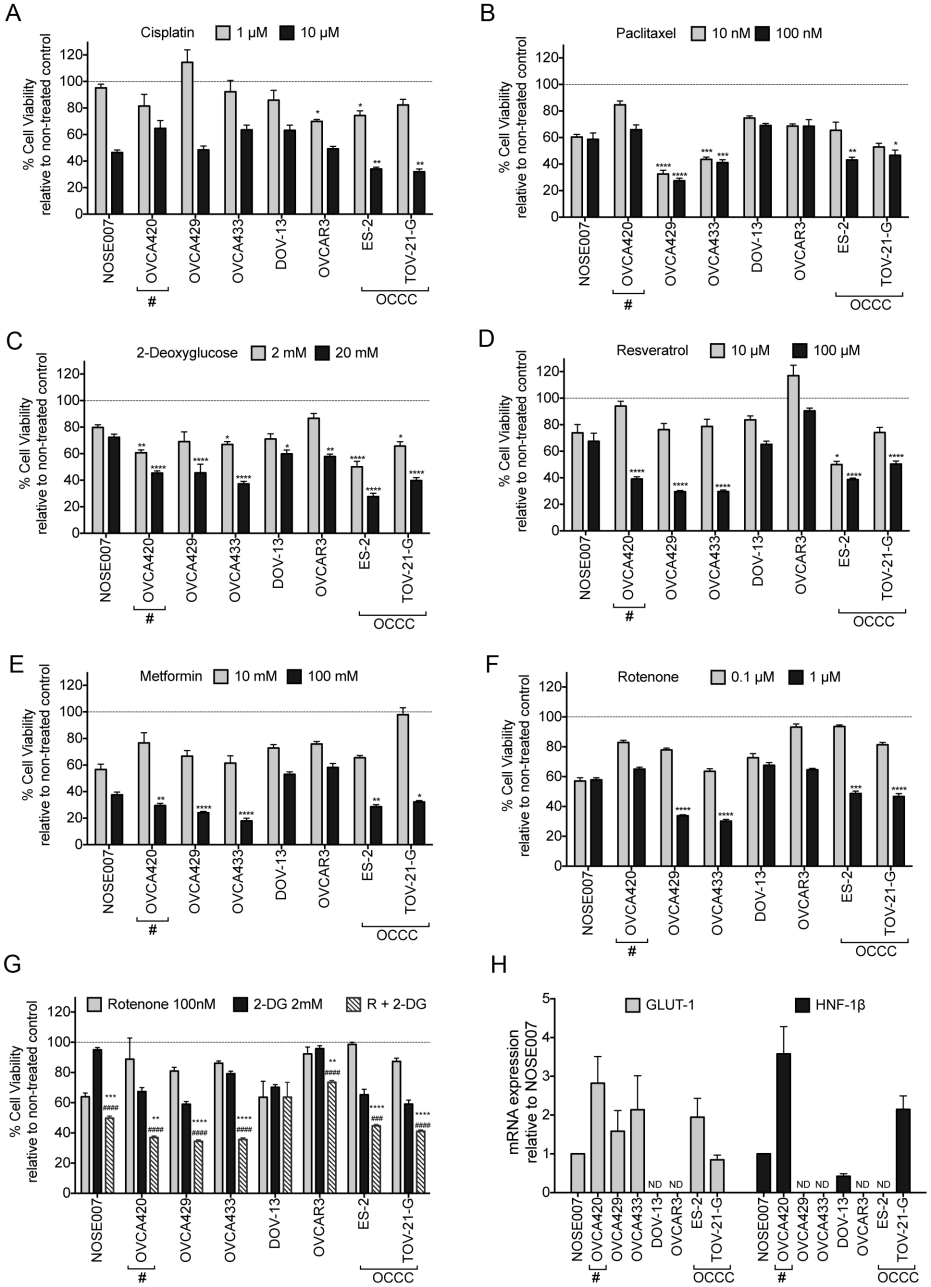
Chemoresistance Profile of Ovarian Cancer cells. Cells were treated with indicated doses for 72**A**. Cisplatin, **B**. Paclitaxel, **C**. 2-Deoxyglucose (2-DG), **D**. Resveratrol, **E**. Metformin, **F**. Rotenone, and **G**. Combination treatment of low dose Rotenone (R; 0.1 µM) and 2-DG (2 mM). Data from one replicative experiment is shown (n = 5-6, A–F: ANOVA, Tukey's post test *p<0.05, **p<0.01, ***p<0.001, ****p<0.0001 significantly less cell viability compared to NOSE007 at same treatment concentration; G: Student's T-test, statistically significant compared to each cell line treatment with rotenone alone [*p<0.05, **p<0.01, ***p<0.001, ****p<0.0001], or 2-DG alone [#p<0.05, ##p<0.01, ###p<0.001, ####p<0.0001]). Asterisks highlight OVCA420 cells, which have defective mitochondrial respiration. **H**. Expression of GLUT-1 and HNF-1β message in Ovarian cancer cells, as assessed by semi-quantitative real time RT-PCR. Expression was correlated to levels in NOSE007 cells (n = 3; ND  =  no measurable signal detected).

Given the identification of different bioenergetics signatures in our set of ovarian cancer cell lines, we wanted to explore if this is predictive of cell line response to chemotherapeutics. Cisplatin and Paclitaxel are commonly used agents in the treatment of ovarian cancer. The development of resistance to Cisplatin is a common feature of ovarian cancer and OCCC has been characterized as a highly cisplatin-resistant histological subtype. Therefore, it was surprising to see a strong decrease in cell viability in ES-2 and TOV-21-G cells in response to this compound (**Figure 4A**). OVCA429 and OVCA433 cells had the greatest response to Paclitaxel, followed by ES-2 and TOV-21G cells (**Figure 4B**). However, there was no clear pattern associated with Cisplatin and Paclitaxel sensitivity and bioenergetics phenotype.

Given the high level of ECAR observed in OCCC cells, it was not surprising that ES-2 and TOV-21-G cells showed highest response to the glycolytic inhibitor 2-deoxy-D-glucose (2-DG; **Figure 4C**). OVCAR3 cells were more resistant to 2-DG, which may be supported by their high variability in ECAR readings (**Figure 3A**). OVCAR3 cells also exhibited slightly higher respiratory reserve than the OCCC cell lines (**Figure 1I**), which may indicate that these cells are able to ramp up their Oxidative phosphorylation in response to glycolysis shut-down more efficiently. Alternative manipulators of glycolysis, including Resveratrol and Metformin resulted in similar cell viability profiles as obtained by 2-DG (**Figure 4D & E**). NOSE007, DOV-13 and OVCAR3 cells were generally more resistant to these compounds. As expected, OVCA420 cells were susceptible to glycolysis inhibition by 2-DG, Resveratrol and Metformin, but this was not markedly different from OVCA429 or OVCA433 cells.

Compared to NOSE007 cells, OVCA429, OVCA433, and the OCCC cell lines ES-2 and TOV-21-G cells had significant reduction in cell viability in response to the mitochondrial inhibitor Rotenone (**Figure 4F**). This may be associated with their high reliance on mitochondrial respiration, which is particularly evident for OVCA429 and OVCA433 cells. NOSE007 and DOV13 cells, which had an overall lower OCR profile were less sensitive to mitochondrial inhibition by Rotenone at a dose of 1 µM. Interestingly, this pattern of Rotenone sensitivity paralleled that of Paclitaxel (**Figure 4B**), suggesting that Paclitaxel may have stronger activity on cells with enhanced mitochondrial respiration. Besides mediating microtubule-dependent effects, Paclitaxel has been shown to elicit its apoptotic effects *via* the mitochondria [25]–[27]. The need for functional mitochondria in eliciting Paclitaxel toxicity was also highlighted by the lack of OVCA420 response to this compound. Cells with increased sensitivity to 2-DG, were more effectively targeted by combination treatment of low dose 2-DG (2 mM) and Rotenone (100 nM), with highest killing observed in in OVCA420, OVCA429, OVCA433 and the two OCCC cell lines ES-2 and TOV-21-G (**Figure 4G**). These data highlight the potential for glycolysis inhibitors and the co-treatment with mitochondrial inhibitors in the therapeutic targeting of highly bioenergetics OCCCs and EOCs that exhibit high OCR.

Alone, bioenergetics profiles may not provide sufficient predictions of chemotherapeutic responses, as is particularly evident in the case of OVCAR3 cells, which also display a highly bioenergetics phenotype, yet were less susceptible to glycolysis and mitochondrial inhibition. However, we did observe a small but significant decrease in OVCAR3 cell viability upon combination treatment of Rotenone and 2-DG (**Figure 4G**).

To assess if glycolysis inhibition was associated with expression patterns of glycolysis regulators, we assessed the levels of the Glucose transporter GLUT-1 and Hepatocyte nuclear factor-1beta (HNF-1β). Expression of GLUT-1 correlated with inhibition of cell viability associated with glycolysis inhibitors 2-DG, Resveratrol and Metformin in the cancer cell lines (**Figure 4H**). Interestingly, GLUT-1 mRNA message was undetectable in DOV-13 and OVCAR3 cells, which were least susceptible to glycolysis inhibition. HNF-1β was recently identified as a potential regulator of the OCCC glycolysis, *via* regulation of GLUT-1 expression [5], [12], [21], [28]. We could only detect message levels of HNF-1β in one of the two OCCC cell lines (TOV-21-G), suggesting that high GLUT-1 levels in ES-2 cells may be regulated by alternate pathways. Interestingly, OVCA420 cells showed highest expression of both HNF-1β and GLUT-1, further highlighting their compensatory increase in glycolysis in response to dysfunctional mitochondrial respiration.

### OVCA20 cells have defective mitochondria and are unable to cope with hypoxic stress

Extracellular flux analysis identified OVCA420 cells to have defective respiratory function (**Figure 1**). This cell line showed significantly lower basal OCR than all other cancer cell lines tested and did not respond to FCCP, suggesting that mitochondrial integrity may be compromised in these cells. OVCA420 cells displayed comparable basal ECAR to other EOC cell lines, and their reliance on glycolysis was demonstrated by a stronger decrease in cell viability, compared to NOSE007 and OVCA433 cells, when cultured in the absence of glucose under normoxic conditions (**Figure S1A**). When cultured under hypoxic conditions both OVCA433 and OVCA429 cells displayed the same glucose dependence (**Figure S1B**). Both OVCA cell lines relied equally on the presence of glutamine in cell culture media, which represents an alternative fuel source for many cancer cells [29].

**Figure 5:**
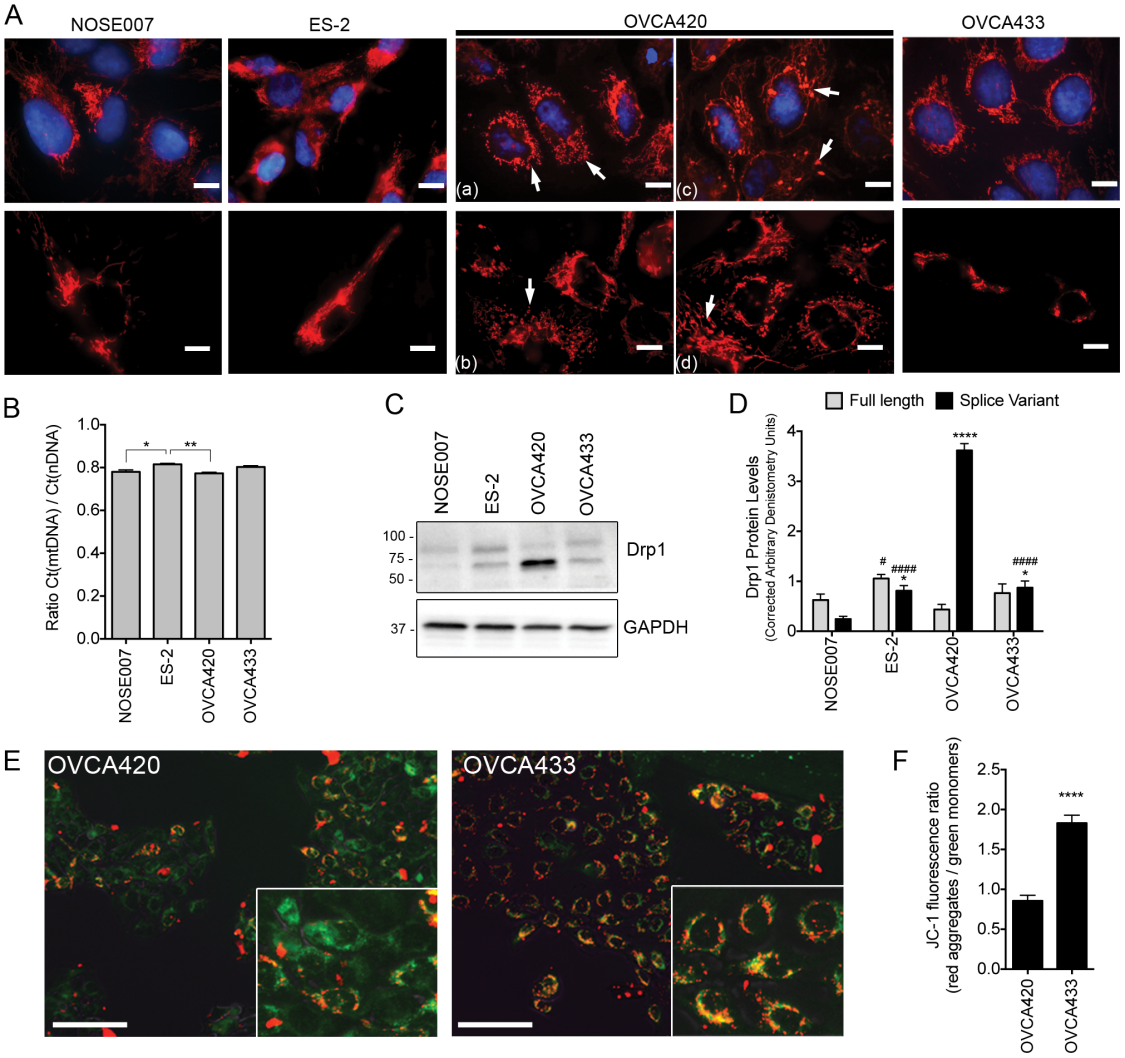
OVCA420 cells have defective mitochondria. **A**. Mitotracker red staining reveals that OVCA420 cells display defects in mitochondrial morphology compared to NOSE007, ES-2 and OVCA433 cells. Representative images show fragmented mitochondria (a&b, arrows) and the presence of large globular aggregates (c&d, arrows) in OVCA420 cells. **B**. Assessment of mitochondrial to nuclear DNA ratios by comparison of mtDNA 16S Ct/nDNA 18S Ct ratios in total DNA from ovarian cancer cell lines, using real time PCR analysis. **C**. OVCA420 cells display enhanced expression of the fission protein Drp1 **D**. Densitometric quantification of the full length and Drp1 splice variant (n = 3, ANOVA, Tukey's post test *p<0.05, ****p<0.0001 significant difference compared to NOSE007; #p<0.05, ####p<0.0001 significant difference compared to OVCA420). **E**. OVCA420 cells display a decrease in mitochondrial membrane potential as evident by the lack of red JC-1 aggregate accumulation and higher staining for JC-1 green monomers. **F**. Quantification of red/green JC-1 staining indicative of membrane potential (n = 35 cells, ****p<0.0001, t-test).

Analysis of Mitotracker Red staining revealed that OVCA420 mitochondrial morphology was highly disordered compared to OVCA433, NOSE007 and ES-2 cells (**Figure 5**). The OCCC cell line displayed strong Mitotracker staining with a vast mitochondrial network extending into all areas of the cell, while NOSE007 and OVCA433 mitochondrial staining was primarily perinuclear. In OVCA420 cells, Mitotracker staining revealed a large number of cells with increased mitochondrial fragmentation (**a, b & d**) and some areas of high Mitotracker aggregation (**Figure 5A** **(d)**), potentially indicating a dysregulation of mitochondrial fission/fusion. Decreased mitochondrial DNA content has been associated with ovarian cancer progression [30], however strong differences in mitochondrial to nuclear DNA ratios were not observed between the cell lines (**Figure 5B**). To determine the potential mechanism for enhanced OVCA420 mitochondrial fragmentation expression of the major protein responsible for mitochondrial fission, Dynamin-Related Protein (Drp1), was assessed [31], [32]. Interestingly, OVCA420 cells displayed significantly higher protein expression of a common Drp1 splice variant (predicted molecular weight: 78 kDa, compared to full length: 82 kDa), which lacks exons 16 and/or 17 in the variable 3′ region of the gene (**Figure 5C&D**) [33], [34]. In addition to changes in mitochondrial morphology OVCA420 cells had significantly decreased JC-1 red fluorescent dye aggregate accumulation in mitochondria, which is indicative of a loss in mitochondrial membrane potential (**Figure 5E&F**). These data suggest that compromised respiration observed by extracellular flux analysis is related to defects in OVCA420 mitochondria.

**Figure 6:**
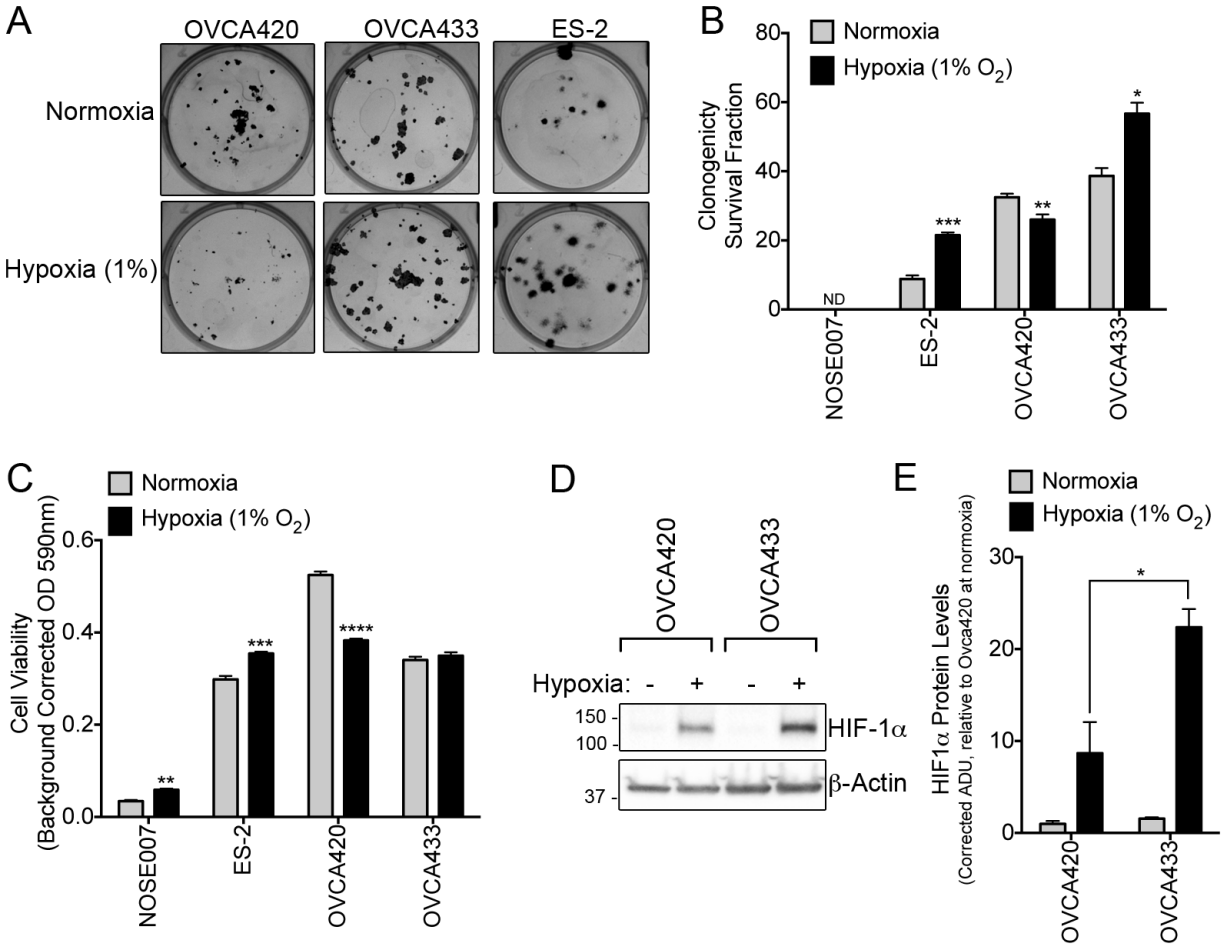
OVCA420 cells with defective mitochondria lack ability to respond to hypoxia. **A**. OVCA420 cells do not increase their clonogenic survival in response to hypoxia, unlike OVCA433 and ES-2 cells. **B**. Clonogenicity was assessed 10 days following incubation at either normoxic (21% O_2_) or hypoxic (1% O_2_) conditions and survival fraction quantified (n = 6, *p<0.05, **p<0.01, t-test). NOSE007 did not form colonies under either condition **C**. OVCA420 cells are unable to maintain cell viability under hypoxic conditions. Cell viability was assessed 72 hours following culturing under normoxic (21% O_2_) or hypoxic (1% O_2_) conditions and assessed by crystal violet uptake assay (n = 6, *p<0.05, t-test). **D**. OVCA420 cells display abrogated HIF-1α protein stabilization in response to hypoxia. Cells were exposed for 2 hours to 1% O_2_ and HIF-1α levels visualized following immediate lysis and immunoblotting. Data were quantified by densitometry analysis relative to actin loading control and normalized to HIF-1α levels observed under normoxia (**E**).

Functionally, we wanted to assess how mitochondrial dysfunction may affect OVCA420 survival and growth. While these cells displayed comparable clonogenic survival and growth rates when compared to other SOC cell lines, their response to hypoxic stress conditions was severely compromised. While OVCA433 and ES-2 clonogenic survival increase in response to hypoxia (1% O_2_), OVCA420 survival was significantly decreased under this stress condition (**Figure 6A & B**). NOSE007 cells were unable to form colonies in this assay. OVCA420 cell viability was also significantly compromised under hypoxic conditions (**Figure 6C**), whereas NOSE007, ES-2 and OVCA433 cells displayed similar or increased cell viability in response to 1% O_2_ culturing. Since mitochondria have been implicated in the regulation of hypoxia *via* HIF-1α, we speculated that OVCA420 may have a compromised HIF-1αresponse. Indeed, HIF-1αstabilization was much lower in OVCA420 in response to 1% O_2_ (**Figure 6D & E**). Using bioenergetic profiling we identified an ovarian cancer cell line with defective mitochondrial function and concomitant abrogation in hypoxic response. This knowledge is imperative for researchers planning to utilize OVCA420 cells for further studies in metabolism and hypoxia.

## Discussion

Through genetic, expression and histological analysis, it is now evident that the classification of ovarian cancer comprises at least 5 distinct diseases. These have been shown to differ significantly in genomic instability, mutation rate and tissue of origin [1]–[3]. In the present study we set out to investigate whether the OCCC can also be distinguished from other EOC subtypes, including the more common SOC, based on bioenergetic profiling. Our data suggest that ovarian cancer cell lines display higher rates of oxidative phosphorylation, compared to a normal epithelial cell line. A distinguishing bioenergetics feature between the histological subtypes was the high ECAR exhibited by OCCC cells, which is an indirect indication of glycolysis. Due to the characterization of OCCC as a stress responsive cancer and this cancer exhibiting high expression of glycolysis-associated genes, we anticipated that OCCC cells rely heavily on glycolysis [12], [15], [35]. It was previously reported that OCCC are more adept at coping with redox and hypoxia-related stress and that they are more sensitive to glucose deprivation-induced cell death [5]. Hepatocyte nuclear factor-1beta (HNF-1β) has been identified as a potential regulator of OCCC stress response and glycolysis. HNF-1β is highly expressed and displays enhanced nuclear localization in most OCCC cases [5], [12], [21]. Further, Okamoto *et al*. recently reported that OCCC cell aerobic glycolysis and glucose uptake *via* GLUT-1 is regulated by HNF-1β [28].

In the present study, reliance of OCCC on glycolysis was further evidenced by the fact that OCCC lines had the highest decrease in cell viability in response to 2-DG, Resveratrol and Metformin. This suggests that targeting glycolysis may be a specific treatment strategy for OCCC. While 2-DG use is not common in ovarian cancer therapy, this compound has been shown to be effective in reducing epithelial ovarian cancer cell viability [36]. In addition, in a progression model of murine ovarian cancer, researchers have shown that late stage ovarian cancer cells are characterized by an increase in glycolytic phenotype and demonstrated effective abrogation of proliferation by sphingosine-mediated inhibition of glycolysis [37]. A number of recent studies have also explored the use of glucose inhibition for treatment against ovarian cancer and identified this to be effective if used in combination therapy [38], or during anaerobic conditions in cisplatin-resistant cells with high hexokinase expression [39]. However, no clinical study has focused on specific use of glycolysis inhibitors for therapeutic intervention of OCCC. Studies such as those by the Konishi group and ours provide evidence for the use of glycolysis inhibition in the treatment of OCCC. One caveat with use of 2-DG in the clinic is its high toxicological profile and limited efficacy. To circumvent this co-treatment of OCCC with platinum-based agents and glycolysis inhibitors might require further exploration. In addition, alternate manipulators of glycolysis including compounds such as resveratrol [40] or the AMPK activator metformin [41] should be investigated for specific use in OCCC, as well as the use of combination therapy targeting mitochondrial respiration and glycolysis.

Interestingly, inhibition of mitochondrial respiration by Rotenone and its effects on cell viability paralleled the response observed with paclitaxel. Besides its effects on microtubules, Paclitaxel has been shown to contribute to apoptosis in a mitochondria-dependent manner. Paclitaxel enhances respiratory rate by opening of the permeability transition pore and induces swelling in isolated mitochondria [25]. It has also been shown to directly interact with mitochondria to enhance cytochrome c release, was demonstrated to decrease Bcl-2 levels and contribute to mitochondria-dependent, but caspase-independent apoptosis [25]–[27]. These data suggest that functional mitochondria may be necessary for complete paclitaxel action, and that these effects are abrogated in cells lacking mitochondrial integrity, such as OVCA420 cells.

In addition to considering glycolysis targeting for therapeutic intervention of OCCC, their bioenergetics signature may provide a tool for improved diagnostics and monitoring of this histological subtype. Compared to SOC, OCCC has been characterized by lower CA125 levels, making this serum marker of ovarian cancer less reliable for OCCC [42]. Considering their reliance on glycolysis, 18F-fluorodeoxyglucose positron emission tomography (FDG-PET) may be useful for initial OCCC tumor detection and monitoring of treatment efficacy and recurrence. FDG-PET has been shown to be useful for detection of recurrent and metastatic EOC, but not borderline lesions, highlighting that glycolysis is enhanced upon tumor progression [43]–[45]. Enhanced uptake of FDG by OCCC tumors was not specifically highlighted in these studies, likely due to the small OCCC patient pool, however this may be a more specific diagnostic tool of benefit to patient populations with this histological subtype.

While OCCC cells showed a high bioenergetics profile, as expected given their previously reported gene expression signature, this profile was not limited to OCCC cells. This suggests that bioenergetics alone may not be a strong predictor of histological subtype. OVCAR3 cells exhibited and overall high bioenergetics profile similar to ES-2 and TOV-21-G cells, albeit ECAR readings being greatly variable between experimental replicates. Further, the OVCAR3 bioenergetics signature did not predict susceptibility to glycolysis and/or mitochondrial inhibition, and these cells were generally the most chemoresistant to metabolic inhibition. This is contrary to a previous study that showed high sensitivity of OVCAR3 cells towards metformin [46], and could be dependent on clonal differences between cell lines of the respective labs. However, we did observe a decrease in OVCAR3 cell viability with combination treatment of 2-DG and Rotenone. OVCAR3 cells did not display significant levels in GLUT-1 and their survival in response to glycolysis and mitochondrial inhibitors requires further investigation, including the potential role for alternate metabolic pathways utilized by these cells. In addition, the validity of OVCAR3 cells as a model for SOC was recently questioned by comparing the cell line's copy number alterations against data from high grade SOC tumor specimen from the Cancer Genome Atlas (TCGA), with the authors concluding that this cell line may not be a true representative of SOC [47].

Given the intricate interplay of cancer cell metabolism, mitochondria and cell signaling, it is imperative to consider bioenergetic profiles in addition to genomic backgrounds when choosing the correct cell lines for *in vitro* and *in vivo* studies. It has been shown that extracellular flux analysis *in vitro* can be correlated to tumor metabolic function *in vivo* [48]. The need for assessing metabolic function was also highlighted by our identification of a SOC cell line with defective mitochondrial oxidative phosphorylation. Supporting this, we showed that mitochondrial morphology and membrane potential was compromised in OVCA420 cells. This not only resulted in abrogated mitochondrial respiration, but a decrease in mitochondria-associated signaling, namely a compromised hypoxic response *via* HIF-1α. The stabilization of HIF-1α has been demonstrated to be reliant on oxygen sensing by mitochondria. While the exact mechanisms of this oxygen sensing remain debated, they include the influence of mitochondrial reactive oxygen species on inhibition of propyl hydroxylase and subsequent stabilization of HIF-1α, as well as the spared oxygen hypothesis, where cellular oxygen (O_2_) levels are controlled by shuttling O_2_ to the mitochondria, which is dependent on proper functioning of electron transport chain complexes [49]–[53].

Our data suggest that OVCA420 cells have defects in mitochondrial fission. A splice variant of the Drp1 fission protein was shown to be highly expressed in OVCA420, which may contribute to increase fission and consequential mitochondrial dysfunction in these cells. The expression of Drp1 splice variants in the 3′ region appears to be common among different cell types. This alternate splicing does not alter the GTPase domain of Drp1 [54], and the levels of expression and role of these splice variants in ovarian cancer has not been investigated. Only recently has a study suggested that Drp1 splice variants may differ in their ability to interact with microtubules [34]. Recent studies have highlighted a role for Drp1 in tumorigenesis. Drp1 expression has been shown to be increased in certain cancers and the use of Drp1 inhibitors proposed as a novel cancer therapeutic intervention [55]. For example, Drp1 expression was significantly enhanced in lung cancer and inhibiting Drp1 with a small molecule significantly inhibited proliferation [56]. Further, Drp1 recruitment to mitochondria and enhanced mitochondrial fission lead to decreased Oxidative phosphorylation and a switch to glycolysis in neuroblastoma [57]. Interestingly, interrogating TCGA data, ovarian cancers displayed the highest gene amplification events for the Drp1 gene DNM1L, among all cancers represented, albeit this alteration only being present in 8% of the samples (data obtained *via* cBioPortal for Cancer Genomics [58], [59]). Further, 11% of high grade SOC tumors displaying increased Drp1 mRNA expression. OVCA420 cells may represent this cohort of ovarian cancers. The role of Drp1 and alterations in mitochondrial fission in ovarian cancer etiology and bioenergetics warrant further investigation.

The present study shows that bioenergetic profiling is a useful tool to characterize different histological subtypes of ovarian cancer in addition to genetic and histological analysis. Extracellular flux analysis might in future allow for bioenergetic screening of ovarian cancer patient tumor sample for validation of histological classification and better determination of treatment strategy. In addition, this analysis can reveal metabolic and mitochondrial defects that can have profound influences on cellular signaling pathways, information that is particularly important for future use of these cell lines as *in vitro* cancer models.

## Supporting Information

Figure S1 **OVCA420 cells rely on glucose under normoxic conditions.** **A**. Cells were cultured in glucose-free and L-glutamine free media, as indicated, for 72 hours. Viability was assessed by crystal violet analysis and expressed as a percentage compared to cells grown in fully supplemented media (2 g/L glucose and 300 mg/L L-glutamine, n = 6, ***p<0.001, t-test). **B**. Cells were cultured with or without media containing glucose for 72 hours in either normoxic (21% O_2_) or hypoxic (1% O_2_) conditions, and cell viability assessed by MTT assay. Viability was expressed as a percentage compared to cells grown in fully glucose supplemented media (2 g/L, n = 6, ****p<0.0001, t-test).
(TIF)
