# Supplementary figures and images for: Bioenergetic Analysis of Ovarian Cancer Cell Lines: Profiling of Histological Subtypes and Identification of a Mitochondria-Defective Cell Line
Source: PLoS One. 2014 May 23;9(5):e98479. doi: 10.1371/journal.pone.0098479 (PMC4032324; doi:10.1371/journal.pone.0098479)

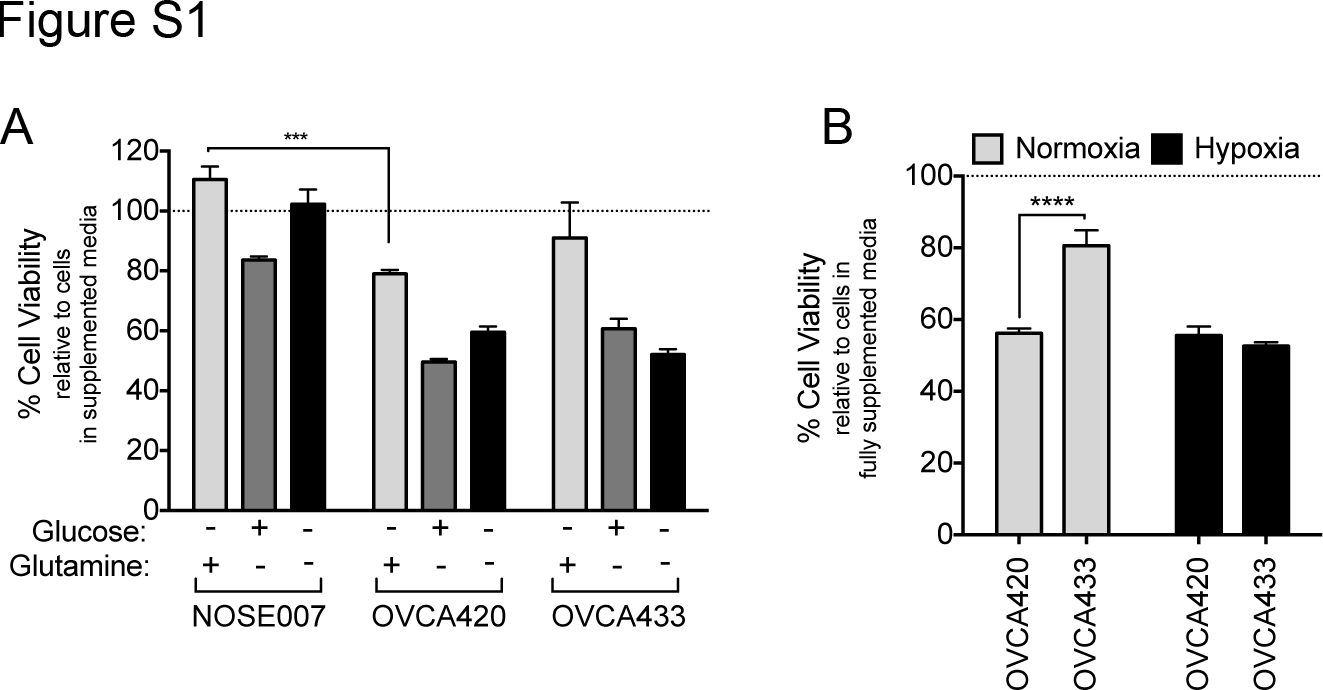

Supplement: Figure S1 — OVCA420 cells rely on glucose under normoxic conditions. A. Cells were cultured in glucose-free and L-glutamine free media, as indicated, for 72 hours. Viability was assessed by crystal violet analysis and expressed as a percentage compared to cells grown in fully supplemented media (2 g/L glucose and 300 mg/L L-glutamine, n = 6, ***p<0.001, t-test). B. Cells were cultured with or without media containing glucose for 72 hours in either normoxic (21% O2) or hypoxic (1% O2) conditions, and cell viability assessed by MTT assay. Viability was expressed as a percentage compared to cells grown in fully glucose supplemented media (2 g/L, n = 6, ****p<0.0001, t-test). (TIF) [file pone.0098479.s001.tif]
